# Poor adherence to antihypertensive medication in patients with hypertension in eastern Sudan: A cross-sectional study

**DOI:** 10.1097/MD.0000000000050736

**Published:** 2026-09-11

**Authors:** Reem A. Younis, Mohamed B. Ali, Ahmed A. Hassan, Ishag Adam, Saeed M. Omar

**Affiliations:** a Department of Internal Medicine, College of Medicine, Qassim University, Buraydah, Saudi Arabia; b Faculty of Medicine, Gadarif University, Gadarif, Sudan; c Faculty of Medicine, Shendi University, Shendi, Sudan; d Department of Obstetrics and Gynecology, College of Medicine, Qassium University, Buraydah, Saudi Arabia.

**Keywords:** adherence, age, associated factors, hypertension, prevalence, Sudan, urban

## Abstract

Adherence to antihypertensives is one of the pillars for controlling hypertension and preventing its complications. Currently, there is no published data on adherence to antihypertensives among hypertensive patients in Sudan. Thus, this study aimed to assess the prevalence of nonadherence to antihypertensive medication and identify the associated factors among hypertensive patients in Gadarif, eastern Sudan. This hospital-based cross-sectional study was conducted in Gadarif, eastern Sudan. Data on demographic characteristics, anthropometry, blood pressure, and clinical history were obtained. Adherence to antihypertensive medication was assessed using the Morisky nedication adherence scale-8. An Morisky medication adherence scale-8 score of <6 was considered nonadherence, and a score of 6 or higher was considered adherence. Multivariate binary regression analysis was performed. A total of 474 adults with hypertension participated in this study; 312 (65.8%) were females. The median age was 53.0 years (interquartile range 45.0–60.0). Of the 474 participants, 446 (94.1%) were nonadherent to their antihypertensive medications, 8 (1.7%) had high adherence, and 20 (4.2%) had medium adherence. Multivariate binary regression analysis indicated that urban residence was associated with nonadherence to antihypertensive medication (adjusted odds ratio = 2.39, 95% confidence interval = 1.06–5.43). Age, sex, education, duration of hypertension, number of medications, income, and presence of comorbidity were not associated with nonadherence to antihypertensive medication. This study highlights a concerning nonadherence rate of 94.1% to antihypertensive medication among hypertensive adults in eastern Sudan during the ongoing war. The findings underscore the significant impact of urban residency on adherence, indicating a need for targeted interventions in these areas. Addressing the barriers to medication adherence is crucial for improving health outcomes in this vulnerable population.

## 1. Introduction

Arterial hypertension is one of the growing health problems, and it was estimated in 2010 that around one-third (31.1%) of the global adult population (1.39 billion people) had hypertension.^[1]^ Hypertension can lead to several morbidities (chronic cardiovascular complications) and all-cause mortality across the globe.^[2,3]^ The prevalence of hypertension is rising globally due to the aging of the population and urbanization changes, such as a change in lifestyle, unhealthy diets (i.e., high sodium and low potassium intake), and reduced physical activity.^[1]^ Patients who are nonadherent to antihypertensive medication experience suboptimal blood pressure control, complications related to hypertension, increased rates of all-cause hospitalization, and higher all-cause mortality, particularly in low- to middle-income countries where both armed conflicts and poor healthcare systems exist.^[4–9]^For example, nonadherence to antihypertensive medication can result in hypertension-related morbidity, such as cardiovascular diseases and stroke.^[9–11]^ Therefore, there is increased concern regarding adherence to antihypertensive medication in many countries, including African ones.^[4,12–17]^ In Africa, varying rates of adherence to antihypertensive medications have been reported.^[13,18–21]^ The determinants of medication adherence included the ability to attain hypertension control, hypertension knowledge, and treatment-related factors, such as belief in the drug’s efficacy, availability of commodities, and sociocultural and financial-related factors.^[13]^ For example, a high prevalence of nonadherence to antihypertensive medication was reported among African hypertensive patients, such as in Ethiopia, at 68.1%.^[16]^ Several factors, such as age,^[17,22,23]^ rural residence,^[22,23]^ being unmarried,^[22]^ low education level,^[17,24]^ family income,^[22]^ alcohol consumption,^[25]^ and number of medications^[23,25]^ and the presence of comorbidity,^[16,24,25]^ were found to be associated with nonadherence to antihypertensive medication among hypertensive patients in African contexts.

While the majority of studies from different countries, including Sudan, addressed nonadherence to antihypertensive medication among hypertensive patients in stable settings,^[22–25]^ few studies were conducted in eastern Sudan.^[16,17]^ No such study explores adherence to antihypertensive medication among hypertensive patients in Sudan. Therefore, such a study is justified by its potential to address urgent public health needs, inform healthcare practices, and contribute valuable knowledge to the field of hypertension management in armed conflict situations. Thus, this study aimed to assess the prevalence of nonadherence to antihypertensive medication and identify the associated factors among hypertensive patients in Gadarif, eastern Sudan.

## 2. Methods

### 2.1. Study area

Gadarif is one of the 18 states in Sudan. The 2008 census estimated the total population of Gadarif State to be around 1,400,000.^[26]^ Situated in eastern Sudan, bordering Ethiopia, Gadarif serves as the capital. The state consists of 11 localities and boasts vast agricultural land, including some of the most significant rain-fed agricultural projects in Sudan. Gadarif State is recognized for its diverse multi-ethnic society, comprising various Sudanese tribes from different regions.^[27]^

### 2.2. Participants and study design

This hospital-based cross-sectional study was conducted from July to October 2024, recruiting hypertensive patients from the referred clinic of the Internal Medicine Unit at Gadarif Public Hospital. Located in the heart of Gadarif city, this hospital serves as a primary healthcare provider for all hypertensive patients in the state. The study adhered strictly to the Strengthening the Reporting of Observational Studies in Epidemiology guidelines.^[28]^ Written informed consent forms were obtained from all participants, indicating their willingness to participate in the study.

### 2.3. Inclusion criteria

All adult patients with hypertension attending the outpatient clinic were invited to participate in the study. Eligible participants were required to meet specific inclusion criteria, including a diagnosis of hypertension for at least 1 year, being prescribed antihypertensive medication, the ability to provide informed consent, and the absence of physical disabilities that could hinder anthropometric assessments.

### 2.4. Exclusion criteria

Pregnant women and individuals who opted not to participate were excluded from the study. Additionally, participants who reported being hypertensive but could not provide evidence of being prescribed medication, as well as those newly diagnosed with hypertension (<1 year), were also excluded.

### 2.5. Sample size calculation

The sample size was calculated using the formula for a single population proportion. The authors estimated that 70% of hypertensive patients were nonadherent to antihypertensive medication during the ongoing war, given the lack of data on this issue among hypertensive patients in Sudan. This estimate was informed by the reported prevalence of nonadherence in Ethiopia, which was 68.1%.^[16]^ Furthermore, based on another Ethiopian study, the authors suggested that hypertensive patients with lower education levels were twice as likely to be nonadherent to antihypertensive medication compared to those with higher education levels.^[24]^ Consequently, a sample size of 474 hypertensive patients was determined, with a 95% confidence level and 80.0% power.

### 2.6. Sampling procedure

In this study, a systematic random sampling method was employed. From the records of hypertensive patients, 1 patient was randomly selected for every 3 patients in consecutive order. If a patient declined to participate or was deemed ineligible, the next patient was chosen to ensure the target sample size of 474 was met.

### 2.7. Data collection

Two trained general practitioners, supervised directly by the research team, gathered sociodemographic information through face-to-face interviews using a questionnaire developed from previous similar studies, including our prior research in eastern Sudan, particularly those conducted during periods of conflict.^[6,16,22,25]^ The sociodemographic data collected comprised age in years, sex (male or female), residence (urban or rural), marital status (married or unmarried), education (low education level or high education level), occupation (employed or unemployed), alcohol consumption (never or current/former), and tobacco use (never or current/former). The questionnaire also gathered detailed information on hypertension history, including its duration, the number of medications taken, and the presence of comorbidities such as diabetes, ischemic heart disease, and renal disease. Participants’ weight and height were measured using standard procedures, and body mass index (BMI) was calculated as weight (kg) divided by height (m^2^).^[29]^

The study utilized the 8-item Morisky medication adherence scale to evaluate participants’ adherence to medication. For the 1st 7 items, responses were scored as “Yes” = 0 and “No” = 1, except for item 5, where the scoring was reversed. The last item employed a 5-point Likert scale with options ranging from “never” to “always.” The primary outcome measured was the adherence status to antihypertensive medication. This Morisky medication adherence scale-8 is commonly used in African contexts.^[22,25]^

### 2.8. Statistics

Data were analyzed using SPSS (IBM Corp.) for Windows (version 22.0). Continuous variables such as age and BMI were evaluated for normality using the Shapiro–Wilk test and were found to be non-normally distributed. Results were reported as proportions or medians with interquartile ranges (IQR). Univariate binary analysis was performed with adherence to antihypertensive medication as the dependent variable, while the independent variables included age, sex, residence, BMI, education, occupation, income, tobacco use (never or current/former), alcohol consumption (never or current/former), duration of hypertension, number of medications, and presence of comorbidities. Variables with a univariate *P* value of <.20 were considered for inclusion in the multivariate binary regression analyses to account for potential confounders. Variables with low frequency, such as alcohol consumption, were excluded from the logistic regression. Adjusted odds ratios and 95% confidence intervals were calculated, with a *P* value of <.05 considered statistically significant.

## 3. Results

### 3.1. General characteristics

A total of 474 adults with hypertension participated in this study, comprising 162 males (34.2%) and 312 females (65.8%). The median age was 53.0 years (IQR: 45.0–60.0), and the median BMI was 26.4 kg/m^2^ (IQR: 24.3–28.7). Among the participants, 67 (14.1%) had comorbidities. Of the 474 individuals, 297 (62.7%) resided in urban areas, 402 (84.8%) were married, and 39 (19.6%) were employed. Additionally, 285 (60.1%) had an education level of at least secondary school, and 209 (44.1%) reported high or moderate income. Most participants, 464 (97.9%), had never consumed alcohol, while 54 (11.4%) had used tobacco. The median duration of hypertension was 5.0 years (IQR: 3.0–7.0), and participants were taking a median of 1 antihypertensive medication (IQR: 1–1). Furthermore, 446 participants (94.1%) were nonadherent to antihypertensive medications; 8 (1.7%) had high adherence, and 20 (4.2%) had medium adherence (Table 1). The majority of nonadherence cases were from urban areas (285 [63.9%] urban vs 161 [36.1%] rural) (Table 2).

**Table 1:** Sociodemographic characteristics of hypertensive patients in eastern Sudan, 2024 (n = 474).

Characteristics		Median	Interquartile range
Age, yr		53.0	45.0–60.0
Body mass index, kg/m^2^		26.4	24.3–28.7
Duration of hypertension		5.0	3.0–7.0
Number of medications		1	1–1
		Frequency	Percentage
Sex	Females	312	65.8
	Males	162	34.2
Marital status	Married	402	84.8
	Unmarried	72	15.2
Residency	Rural	177	37.3
	Urban	297	62.7
Education status	≥Secondary level	285	60.1
	<Secondary level	189	39.9
Occupation status	Unemployed	381	80.4
	Employed	93	19.6
Presence of comorbidity	No	407	85.9
	Yes	67	14.1
Tobacco use	Never	420	88.6
	Current/former	54	11.4
Alcohol consumption	Never	464	97.9
	Current/former	10	2.1
Income	High/moderate	209	44.1
	Low	265	55.9
Adherence to antihypertensive medication	High adherence	8	1.7
	Medium adherence	20	4.2
	Nonadherence	446	94.1

**Table 2:** Univariate binary analysis for factors associated with nonadherence to antihypertensive medication among hypertensive patients in eastern Sudan during the ongoing war, 2024.

Variable		Nonadherence (n = 446)	Adherence (n = 28)	Odds ratio	95% confidence interval	*P* value
		Median (interquartile range)				
Age, yr		52.0 (45.0–59.0)	60.5 (49.7–68.7)	0.92	0.89–96	<.001
Body mass index, kg/m^2^		26.4 (24.3–28.7)	25.7 (22.3–30.1)	1.01	0.91–1.12	.792
Duration of hypertension		4.0 (3.0–7.0)	5.0 (3.0–11.0)	0.89	0.81–0.97	.012
Number of medications		1.0 (1.0–1.0)	1.5 (1.0–4.0)	0.63	0.51–0.78	<.001
		Frequency (percentage)				
Sex	Males	148 (33.2)	14 (50.0)	Reference		
	Females	298 (66.8)	14 (50.0)	2.01	0.93–4.33	.074
Marital status	Married	377 (84.5)	25 (89.3)	Reference		
	Unmarried	69 (15.5)	3 (10.7)	1.52	0.44–5.19	.499
Residency	Rural	161 (36.1)	16 (57.1)	Reference		
	Urban	285 (63.9)	12 (42.9)	2.36	1.10–5.11	.029
Education status	≥Secondary	263 (59.0)	22 (78.6)	Reference		
	<Secondary	183 (41.0)	6 (21.4)	2.55	1.01–6.41	.047
Occupation status	Unemployed	356 (79.8)	25 (89.3)	Reference		
	Employed	90 (20.2)	3 (10.7)	2.10	0.62–7.13	.231
Presence of comorbidity	No	390 (87.4)	17 (60.7)	Reference		
	Yes	56 (12.6)	11 (39.3)	0.22	0.09–0.49	<.001
Tobacco use	Never	396 (88.8)	24 (85.7)	Reference		
	Current/former	50 (11.2)	4 (14.3)	0.75	0.25–2.27	.620
Alcohol consumption	Never	438 (98.2)	26 (92.9)	Reference		
	Current/former	8 (1.8)	2 (7.1)	0.23	0.04–1.17	.078
Income	High/moderate	201 (45.1)	8 (28.6)	Reference		
	Low	245 (54.9)	20 (71.4)	0.48	0.21–1.13	.094

### 3.2. Factors associated with nonadherence to antihypertensive medication

In the univariate binary regression analysis, a significant association was found between nonadherence to antihypertensive medication and several factors, including age, duration of hypertension, number of drugs, residence, education, and presence of comorbidities. In contrast, sex, BMI, marital status, occupational status, income, tobacco use, and alcohol consumption showed no association with nonadherence to antihypertensive medication (Table 2).

Multivariate binary regression analysis indicated that urban residence was associated with nonadherence to antihypertensive medication (adjusted odds ratio = 2.39, 95% confidence interval = 1.06–5.43). Age, sex, education, duration of hypertension, number of medications, income, and presence of comorbidity were not associated with nonadherence to antihypertensive medication (Table 3).

**Table 3:** Multivariate binary analysis for factors associated with nonadherence to antihypertensive medication among hypertensive patients in eastern Sudan during the ongoing war, 2024.

Variable		Adjusted odds ratio	95% confidence interval	*P* value
Age, yr		0.95	0.91–1.01	.095
Duration of hypertension		0.97	0.88–1.08	.653
Number of medications		0.85	0.61–1.19	.355
Sex	Male	Reference		
	Female	0.54	0.22–1.29	.168
Residence	Rural	Reference		
	Urban	2.39	1.06–5.43	.037
Education status	≥Secondary	Reference		
	<Secondary	1.19	0.40–3.55	.744
Presence of comorbidity	No	Reference		
	Yes	0.64	0.17–2.2.2	.493
Income	High/moderate	Reference		
	Low	0.78	0.30–2.06	.627

## 4. Discussion

The findings of this study reveal a high nonadherence rate of 94.1% in this population, with urban residents being at a higher risk of nonadherence. The prevalence of nonadherence is notably higher than the nonadherence rate (30%) reported in eastern Sudan,^[6]^ in Ethiopia, 68.1%,^[16]^ in Eretria, 72.8%,^[20]^ and in Tanzania, 23.1%.^[21]^ In their recent (2023) meta-analysis, which enrolled 10 studies, Shin et al reported that the overall prevalence of antihypertensive adherence in Africa was 34.1%.^[13]^

The high rate of nonadherence to antihypertensive drugs could be explained by several factors, among which is the ongoing war in Sudan. It has been previously reported that war could influence adherence to antihypertensive medications.^[16,17,30]^ A hospital-based cross-sectional study conducted in the war-torn region of Ethiopia during times of armed conflict included 417 participants, showing that the degree of medication nonadherence was 68.1% among chronic patients.^[16]^ Another study in Ethiopia revealed that the war in Tigray has caused significant disruptions in healthcare services, resulting in a high rate of loss to follow-up for patients with chronic diseases, including hypertension.^[30]^ In Ukraine, a 4-month follow-up study including 1299 hypertensive patients during the war found that, in the 1st month, 50 patients (3.8%) either stopped or did not begin antihypertensive therapy; this figure rose to 71 patients (5.5%) in the 2nd month and 127 patients (9.8%) in the 3rd month.^[17]^ The ongoing war in Sudan has resulted in several devastating effects on the healthcare systems.^[31–33]^ Hemmeda et al reported that the ongoing war has severely affected the already deteriorating pharmaceutical situation in Sudan, leading to a significant decline in healthcare services.^[31]^ Hassan et al found that the ongoing war in Sudan has significantly affected the management of hypertension, worsening the condition due to chronic stress, disrupted healthcare services, and lifestyle changes.^[32]^

This study showed that urban resident hypertensive patients were >2 times more likely to experience nonadherence to antihypertensive medication compared to rural ones. Likewise, Magnabosco et al reported a higher prevalence of nonadherence in urban areas (63.4%) compared to rural ones in Brazil (61.9%).^[34]^ In contrast, other studies in Ethiopia and Saudi Arabia reported that rural residents were at risk of nonadherence to antihypertensive medication.^[22,23,35]^ The urban nonadherence to antihypertensive medication observed in this study could be attributed to several interconnected factors. Urban hypertensive patients may exhibit higher rates of nonadherence to antihypertensive medication compared to their rural counterparts, particularly in the context of increased displacement in eastern Sudan.^[36,37]^ Firstly, urban environments often present more significant lifestyle stressors, including overcrowding, economic instability, and social disintegration, which can negatively impact mental health and motivate patients to prioritize immediate survival over chronic disease management. Secondly, access to healthcare services can be fragmented in urban areas, particularly during times of conflict.^[36,38]^ Displaced individuals may face challenges in obtaining medications due to supply shortages, disrupted healthcare facilities, or financial constraints, which can hinder consistent medication adherence.^[36,38]^ Moreover, the rapid influx of displaced populations into urban settings can overwhelm existing healthcare systems, resulting in longer wait times and reduced patient-provider interactions.^[36,38]^ This may diminish opportunities for education on the importance of medication adherence. Consequently, the combination of these factors contributes to a higher likelihood of nonadherence among urban hypertensive patients compared to those in rural areas, where community support and resources may be more stable. On the other hand, rural hypertensive patients may be less likely to be nonadherent to antihypertensive medication due to stronger community ties, more stable healthcare access, and lower stress levels compared to urban counterparts. Additionally, rural settings often provide a greater sense of continuity in care, fostering adherence despite the challenges posed by displacement.

In contrast to this study, other studies have identified age,^[17,22,23]^ being male,^[34]^being unmarried,^[22]^ low education level,^[17,24]^ family income,^[22]^ alcohol consumption,^[25]^ number of medications,^[23,25]^ and the presence of comorbidity,^[16,24,25]^ as factors associated with nonadherence to antihypertensive medication among hypertensive patients, especially in African contexts. Researchers should consider these conflicting findings as an opportunity to assess their own countries or regions and develop localized solutions, particularly during periods of armed conflict or war.

### 4.1. Strengths and implications of the study

This study had several strengths and implications. The study’s strengths included: First, timely and relevant focus, as it addresses a critical public health issue during an ongoing war. This relevance enhances the urgency and importance of the findings. Second, identification of associated factors: By examining various factors related to nonadherence, the study contributes to a better understanding of the complexities surrounding medication management in hypertensive patients, which can inform targeted interventions. Third, baseline data for future research: the findings establish a baseline prevalence of nonadherence in a unique context, which can serve as a reference point for future studies assessing the impact of interventions or changes in the healthcare system in conflict-affected areas. The implications included: First, health policy development: the high prevalence of nonadherence underscores the need for policymakers to develop strategies that specifically address the barriers to medication adherence in conflict settings, such as improving access to medications and healthcare services. Second, targeted interventions: the identification of associated factors can guide the design of targeted interventions aimed at improving medication adherence among hypertensive patients, including educational programs and support systems tailored to the unique challenges faced by individuals in conflict zones. Third, future research directions: the study highlights the necessity for further research into the long-term effects of armed conflict on chronic disease management. Future studies could explore the sustainability of adherence interventions and the impact of socio-political changes on health behaviors in similar contexts.

### 4.2. Limitations of the study

Despite the strengths mentioned above, this study has several limitations that need to be acknowledged. Additionally, suggestions are provided for overcoming these limitations in future studies. First, selection bias: the study’s hospital-based design may not accurately represent the entire population of hypertensive patients, as individuals who seek care may differ from those who do not. Therefore, future studies could employ a multicenter approach, including rural and urban clinics, to capture a more diverse patient population. Additionally, utilizing community surveys could help include individuals who do not visit hospitals. Second, the cross-sectional design: the cross-sectional nature of the study limits the ability to infer causality between factors and nonadherence to medication. Therefore, future research could utilize a longitudinal design to track changes in medication adherence over time. This approach would provide insights into how adherence behaviors evolve, particularly in response to the ongoing war. Third, self-reported adherence: the reliance on self-reported data may lead to inaccuracies due to recall bias or social desirability bias, which can affect the validity of the reported adherence rates. Therefore, future studies could incorporate objective measures of adherence, such as drug measurement, medication possession ratios, or electronic pill monitoring, to complement self-reported data.^[39,40]^ Additionally, including qualitative interviews could provide deeper insights into the reasons behind nonadherence.

## 5. Conclusion

This study highlights a concerning nonadherence rate of 94.1% to antihypertensive medication among hypertensive adults in eastern Sudan during the ongoing war. The findings underscore the significant impact of urban residency on adherence, indicating a need for targeted interventions in these areas. Addressing the barriers to medication adherence is crucial for improving health outcomes in this vulnerable population. Future research should focus on understanding the underlying factors contributing to nonadherence and developing context-specific strategies to enhance medication compliance, ultimately improving hypertension management amidst the challenges posed by the ongoing war.

## Acknowledgments

We would like to thank the study participants for their sincere cooperation and the provision of valuable information.

## Author contributions

**Conceptualization:** Reem A. Younis, Mohamed B. Ali, Saeed M. Omar.

**Data curation:** Ahmed A. Hassan, Reem A. Younis, Mohamed B. Ali, Saeed M. Omar.

**Formal analysis:** Ishag Adam.

**Methodology:** Reem A. Younis, Mohamed B. Ali, Ishag Adam.

**Supervision:** Mohamed B. Ali, Saeed M. Omar.

**Project administration:** Ishag Adam.

**Validation:** Ishag Adam.

**Writing – original draft:** Ahmed A. Hassan, Reem A. Younis, Mohamed B. Ali, Ishag Adam, Saeed M. Omar.

**Writing – review & editing:** Ahmed A. Hassan, Reem A. Younis, Mohamed B. Ali, Ishag Adam, Saeed M. Omar.
